# A Unified YOLOv8 Approach for Point-of-Care Diagnostics of Salivary *α*-Amylase

**DOI:** 10.3390/bios15070421

**Published:** 2025-07-02

**Authors:** Youssef Amin, Paola Cecere, Pier Paolo Pompa

**Affiliations:** 1 Istituto Italiano di Tecnologia (IIT), Nanobiointeractions & Nanodiagnostics, Via Morego 30, 16163 Genova, Italy; paola.cecere@iit.it; 2 College of Optical Science and Engineering, Zhejiang University, Hangzhou 310058, China

**Keywords:** α-Amylase, convolutional neural networks (CNNs), edge computing, image segmentation, machine learning, object detection, point-of-care testing, YOLOv8

## Abstract

Salivary α-amylase (sAA) is a widely recognized biomarker for stress and autonomic nervous system activity. However, conventional enzymatic assays used to quantify sAA are limited by time-consuming, lab-based protocols. In this study, we present a portable, AI-driven point-of-care system for automated sAA classification via colorimetric image analysis. The system integrates SCHEDA, a custom-designed imaging device providing and ensuring standardized illumination, with a deep learning pipeline optimized for mobile deployment. Two classification strategies were compared: (1) a modular YOLOv4-CNN architecture and (2) a unified YOLOv8 segmentation-classification model. The models were trained on a dataset of 1024 images representing an eight-class classification problem corresponding to distinct sAA concentrations. The results show that red-channel input significantly enhances YOLOv4-CNN performance, achieving 93.5% accuracy compared to 88% with full RGB images. The YOLOv8 model further outperformed both approaches, reaching 96.5% accuracy while simplifying the pipeline and enabling real-time, on-device inference. The system was deployed and validated on a smartphone, demonstrating consistent results in live tests. This work highlights a robust, low-cost platform capable of delivering fast, reliable, and scalable salivary diagnostics for mobile health applications.

## 1. Introduction

Salivary diagnostics has emerged as a promising, non-invasive alternative to traditional blood-based biomarker detection, offering a convenient and accessible method for monitoring physiological and pathological conditions [1], as well as providing insight into certain disease states and responses to treatment [2]. Saliva contains a diverse range of biomarkers, including hormones, interleukins, albumin, DNA, mRNA, antioxidants, and enzymes, which reflect various biological processes and health conditions [3,4,5,6,7].

Among various salivary biomarkers, α-amylase is recognized as a reliable and non-invasive indicator of autonomic nervous system (ANS) activity, particularly in response to physical and psychological stressors [8,9,10,11,12]. Salivary α-amylase (sAA) levels have been shown to increase under exercise-induced stress (e.g., running [13], bicycle tests [14]) and psychosocial challenges [15,16,17], as well as to decrease during relaxation interventions [18], highlighting its role in sympathetic nervous system (SNS) activation [1,16] and its potential as a valuable biomarker for stress monitoring. Besides stress-related applications, sAA has also been linked to metabolic conditions, including obesity and insulin resistance [19,20].

Traditionally, sAA is measured using laboratory-based enzymatic activity assays, including spectrophotometric [21], fluorometric [22], and colorimetric [23,24,25,26] techniques. Some commercial kits utilize a starch-based substrate that, upon cleavage by amylase, releases a chromogenic product measurable via absorbance, typically at 405 nm [27]. While these methods provide high sensitivity and reliability, they are time-consuming, require laboratory environments, expensive equipment, trained personnel, and often involve sample transportation and storage under refrigeration [9,16,28]. These constraints hinder their utility in real-time, field-based, or self-administered stress monitoring applications. Additionally, other traditional colorimetric tests often depend on visual interpretation of the color by users, which introduces variability due to differences in color perception and environmental lighting conditions [29,30].

To address these limitations, several portable and paper-based biosensors have been proposed for point-of-care testing (POCT). In this context, Yamaguchi et al. introduced an amylase activity monitor consisting of a test strip, a saliva transcription device, and an optical analyzer, enabling rapid semi-quantitative detection of sAA [23]. However, the accuracy of the measurements was found to be highly sensitive to variations in reaction time, where ±6 s of deviations could cause up to 30% error. More recently, smartphone-integrated biosensing platforms have emerged, utilizing built-in processing and imaging capabilities. Zhang et al. developed a potentiometric smartphone-based sensor that detects sAA through an electrochemical reaction involving starch and a redox mediator, yielding results within five minutes [28]. While promising, these approaches still require calibration, sample preprocessing, and controlled reaction conditions (such as pH, temperature, and sample viscosity) [16] and may show non-linear behavior at high enzyme concentrations due to substrate saturation [24]. Such factors highlight the need for innovative approaches that provide rapid, reproducible, and easy-to-interpret results. Ideally, such methods should be integrated into smart point-of-care healthcare devices that are fully automated and capable of extracting meaningful information from complex biological patterns.

In this context, AI-driven saliva diagnostics has demonstrated the potential of machine learning (ML) in non-invasive disease detection and assessment [31,32,33,34,35]. Additionally, recent advancements in AI and computer vision have facilitated the development of automated POC diagnostic systems that can analyze biological samples using image-based processing techniques. AI offers the opportunity to obtain direct classification of the colorimetric reaction outcomes. Specifically, deep learning-based computer vision enables the interpretation of color intensity from colorimetric tests [36,37,38,39,40]. This has been applied in various diagnostic domains, from heavy metal detection to clinical biomarker monitoring. Our previous work has also explored the application of computationally light deep learning algorithms for real-time classification on embedded systems, highlighting the feasibility of deploying efficient neural networks in resource-constrained environments [41]. However, integrating deep learning models into smartphone-based point-of-care systems presents several challenges. Many studies have reported issues such as the need for large labeled datasets, high computational requirements, sensitivity to lighting variability, and difficulties in classifying closely spaced concentration levels [42,43,44,45]. For instance, models trained on RGB or HSV color spaces often require the addition of reference color patches or preprocessing pipelines to standardize inputs [46,47]. Moreover, classifiers like SVM or DNNs, while powerful, can face overfitting and dimensionality issues when applied to small, heterogeneous biological datasets [48,49]. Cloud-based processing approaches have been proposed to overcome mobile hardware limitations, but these are often unsuitable for real-time or offline operation in resource-limited environments [50,51,52]. These limitations emphasize the need for lightweight, efficient, and robust architectures, such as mobile-friendly object detection models, that can reliably analyze colorimetric assays without dependence on external hardware or intensive preprocessing.

Our previous work introduced a smartphone-integrated system for antioxidant detection in saliva using a two-stage YOLOv4-tiny and CNN architecture, achieving high classification accuracy for a five-class colorimetric problem [33]. While this modular pipeline proved effective, demonstrating a powerful yet modular framework for mobile point-of-care testing, it introduced additional architectural complexity due to sequential processing and was limited in class granularity. Building on this framework, the present study focuses on a more challenging biomarker (salivary α-amylase) requiring finer classification granularity and improved model efficiency.

In this work, to standardize the image acquisition process, we introduce SCHEDA (Smart Colorimetric Hardware for Enhanced Diagnostic Application), a portable, smartphone-compatible imaging platform designed for POC applications. By incorporating fixed illumination, a light-isolated imaging chamber, and consistent sample positioning, SCHEDA ensures uniform imaging conditions, thereby mitigating the influence of external lighting and reflections. This hardware-level standardization enables robust and reproducible colorimetric analysis across diverse usage scenarios and supports the reliable performance of downstream AI models. Furthermore, we propose a deep learning-based approach for the automated classification of α-amylase concentration in saliva samples using image analysis. Specifically, our method combines the hardware platform SCHEDA with a lightweight AI pipeline to achieve high accuracy in colorimetric analysis. The device is wirelessly controlled via a smartphone application using Bluetooth Low Energy (BLE), allowing for image acquisition and data transmission to any smartphone while maintaining consistent imaging conditions. This decouples hardware-based image capture from the variability of mobile device cameras, ensuring reproducibility across diverse environments and user devices. The acquired images are then processed using a deep learning pipeline, where we compare two distinct approaches to classify eight color intensity categories corresponding to different α-amylase concentrations:

- **YOLOv4-CNN (Two-Block Approach)**: A previously established pipeline [33] that utilizes YOLOv4 for object detection to localize the saliva sample, followed by a CNN classifier to determine the color intensity and corresponding biomarker concentration.
- **YOLOv8 (Proposed One-Block Approach)**: A unified model where YOLOv8 performs simultaneous segmentation and classification in a single step, aiming to improve efficiency and accuracy.

The main contribution of this study is to demonstrate the superiority of YOLOv8 over the YOLOv4-CNN pipeline in terms of classification accuracy, model simplicity, and deployment efficiency. It ensures that classification is based only on precisely segmented pixels, improving accuracy while also reducing computational overhead and model size. Furthermore, the development and integration of a custom-built, app-controlled imaging device (SCHEDA) provides a scalable, cost-effective, and reproducible platform for smart, field-deployable diagnostics. This YOLOv8 pipeline simplified the architecture, reduced computational load, and enabled on-device real-time inference, highlighting its potential for rapid, accurate, and accessible point-of-care diagnostics.

The structure of this paper is as follows: Section 2 details the methodology, Section 3 presents the experimental setup, Section 4 presents the results and discussion, and Section 5 provides the concluding remarks.

## 2. Methodology

### 2.1. Imaging Setup for Consistent Image Quality

For color intensity classification problems, it is important to maintain controlled data collection conditions such as ambient light, illumination variance, and camera optics. Any variations in these conditions will result in a change in the color representation, which will adversely affect the performance of the ML models, especially in applications where the model has to classify shades of a specific color to predict the correct concentration level of the biomarker (i.e., α-amylase). To address these challenges, we developed SCHEDA, a custom-designed imaging device that ensures standardized optical conditions for saliva sample analysis. It integrates a low-cost camera module, a precisely controlled LED illumination system, and a sample positioning mechanism to minimize lighting variability and reflections. The system is powered by an ESP32-S3-WROOM microcontroller (Espressif Systems, Shanghai, China), which manages image acquisition, illumination control, and wireless data transmission to a smartphone application via BLE. By maintaining uniform imaging conditions for the training and testing datasets, SCHEDA should allow us to enhance the reliability and accuracy of ML/DL models in classifying subtle color variations associated with different α-amylase concentrations. Further details on the hardware architecture and operational workflow of SCHEDA are discussed in a subsequent section.

### 2.2. Assessing α-Amylase in Saliva

Our colorimetric test evaluates the activity of α-amylase in saliva samples using a dye-based method that exploits the enzyme ability to hydrolyze α-glycosidic bonds in polysaccharides [24,53]. Specifically, we employed 2-chloro-4-nitrophenyl-α-D-maltotrioside as a chromogenic substrate. Upon enzymatic cleavage by salivary α-amylase, this substrate releases 2-chloro-4-nitrophenol, a yellow-colored dye [24,54,55]. Within minutes, the reaction solution transitions from transparent to yellow, with the intensity of the color directly correlating to the α-amylase concentration in the sample. The method was validated using a spectrophotometric assay kit (Abcam, Cambridge, UK, amylase assay kit), which monitors the increase in absorbance at 405 nm over time. This allowed for accurate quantification of enzyme activity and confirmed that our test could reliably measure physiological levels of salivary amylase. To adapt the colorimetric test for POC use, we optimized the reaction by incorporating maltose as a non-competitive inhibitor and fine-tuning the substrate-to-inhibitor ratio. This calibration ensured that the dynamic range of color development (from transparent to intense yellow) matched the expected physiological range of α-amylase concentrations.

In our POC test, we aim to distinguish between different sAA concentration levels. Therefore, unstimulated saliva samples were collected from healthy donors instructed to avoid food, drink, and oral hygiene procedures for at least 30 min prior to collection. The saliva was collected and stored at room temperature and used without any pretreatment. For each trial, the vial containing the reaction mixture was placed in the SCHEDA device, and images were acquired every 30 s for 10 min. During the reaction, a yellow color is developed at different rates depending on the α-amylase concentration, shifting from nearly transparent to distinct shades of yellow. All saliva samples were collected from adult volunteers after obtaining informed consent, in accordance with the ethical guidelines of the Istituto Italiano di Tecnologia (IIT), and the use of saliva was approved by the Ethical Committee of Regione Liguria (405/2020-DB id 10787).

**Figure 1:**
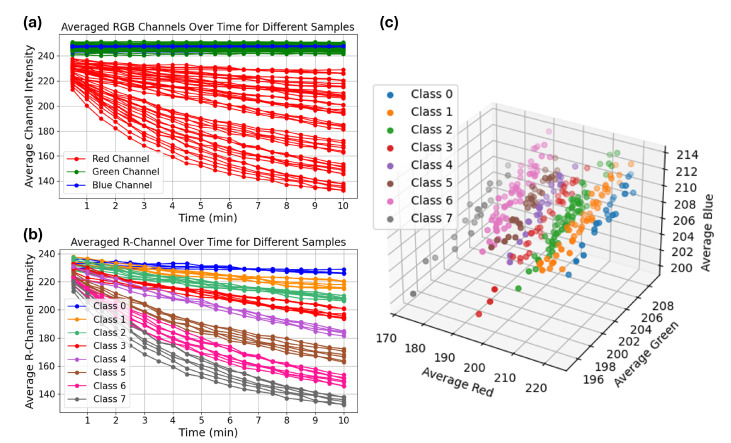
RGB intensity analysis of the colorimetric reaction. (**a**) Channel-wise intensity over time. (**b**) Red channel kinetics for each α-amylase class. (**c**) RGB color space distribution of samples after 10 min divided to 8 classes.

To quantitatively analyze the kinetics of color change, a region of interest (ROI) within the vial was manually cropped from each captured image and used to extract the average RGB intensity values. Figure 1a shows the average intensity of red, green, and blue channels over time for different samples. As illustrated, the red channel intensity decreased significantly over time, while the green and blue channels remained relatively stable. This suggests that the red channel carries the most discriminative information related to the yellow color development. To evaluate class-wise trends, we computed the average red channel intensity curves for all eight classes. Figure 1b displays the kinetics of red channel intensity over time, grouped by α-amylase class. The data show that higher α-amylase concentrations (e.g., Classes 5–7) result in a faster drop in red intensity, indicating rapid color development, whereas lower concentrations (Classes 0–2) show a slower and more gradual intensity decrease. Similarly, Figure 1c displays the average RGB intensities of a larger set of samples captured at the 10-min mark, visualized in the RGB color space. Each point represents a sample, color-coded by its corresponding α-amylase class. The plot reveals that samples with different concentration levels tend to cluster in distinct regions, particularly along the red channel axis, which further highlights the discriminative power of red channel intensity.

**Figure 2:**
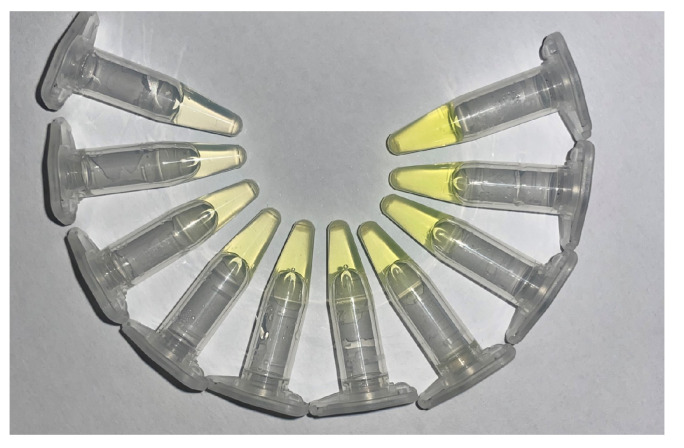
Representative images of vial samples corresponding to different α-amylase concentrations, illustrating the distinct color intensities observed at time = 10 min.

This time-resolved analysis was particularly important for identifying the optimal window for classification, as class-wise separation increased over time. By 10 min, RGB intensity values, especially in the red channel, were clearly distinguishable across classes, with minimal overlap. This motivated our decision to use t = 10 min images for training machine learning models, as this frame provided the highest inter-class separability. Finally, visual observation supported the quantitative findings. As shown in Figure 2, the vials at 10 min exhibited a clear yellow gradient corresponding to increasing α-amylase concentrations (from the left to the right), reinforcing the relationship between enzyme level and color intensity development.

### 2.3. Data Collection

We established a systematic data collection protocol in which saliva samples were collected from multiple donors and colorimetric data were acquired using the SCHEDA device under standardized conditions. For each sample, an image of the colorimetric reaction was captured at t=10 min using the device, ensuring consistency across all samples. To facilitate the data acquisition process, we developed a Python-based interface (Python 3.11), that enables wireless communication with the SCHEDA device via Bluetooth. The script automates the process of sending commands to the device, instructing it to capture an image and subsequently receiving the transmitted image for storage and further processing.

### 2.4. Data Augmentation

The data collection process for this study was both time-consuming and costly, as each saliva sample had to be quantified using a commercial kit to determine its α-amylase concentration. Additionally, for each colorimetric test, images were captured 10 min after test initiation. These constraints significantly limited the number of samples that could be collected within a reasonable timeframe, resulting in a labeled dataset of 480 sample images, with a slight class imbalance across the eight classes.

This class imbalance creates a challenge for model training, as underrepresented classes could lead to biased predictions and reduced generalization. A dataset of this scale increases the risk of overfitting if not managed properly. Therefore, to effectively train an ML model to solve an 8-class classification problem with high accuracy, we had to increase the size of the dataset.

To address these challenges, data augmentation was applied not only to expand the dataset but also to balance the class distribution. Augmentations such as flipping, scaling, and shearing were selectively applied, ensuring a more balanced distribution across different α-amylase concentration levels. This targeted augmentation strategy helped mitigate the class imbalance while preserving the essential color characteristics of the samples.

To prevent data leakage and ensure a fair evaluation of the model’s generalization ability, the dataset was first split into training, validation, and test sets before applying data augmentation. This step was necessary to avoid situations where an augmented version of an image in the training set would appear in the test set, which could lead to overestimated model performance. Only the training set underwent augmentation, ensuring that the validation and test sets consisted of original, unaltered images.

As a result, the dataset size increased from 480 to 1024 images, with some images within some classes requiring multiple augmentation techniques to achieve balance. The augmented dataset provided a more robust training set that would improve the model’s ability to generalize across different α-amylase concentration levels. A detailed breakdown of the number of samples per class before and after augmentation is presented in Table 1.

### 2.5. Convolutional Neural Network

Convolutional Neural Networks (CNNs) are one of the most popular deep neural networks used in a multitude of applications. CNNs are composed of different building blocks, such as convolutional, pooling, and fully connected layers. In this study, we adopted a one-dimensional Convolutional Neural Network (1D-CNN) architecture for classifying α-amylase concentration levels from colorimetric images. This model is an adaptation of the 2D CNN architecture previously employed for colorimetric classification tasks [33] and builds upon the success of 1D-CNNs in other classification domains [56].

As discussed above, rather than using the full RGB image as input, we selected only the red channel, as it contains the most discriminative information for distinguishing color intensity related to α-amylase concentration. The extracted red channel was passed directly to the CNN as a 2D array, and a 1D convolutional layer was applied along one spatial dimension to perform feature extraction. This approach reduced model complexity while preserving essential information, enabling efficient and accurate classification using only a single color channel.

The architecture retains the same core design principles as the original 2D CNN. It is composed of a series of functional blocks, each including a 1D convolutional layer with ReLU activation, followed by a dropout layer and average pooling layer. These blocks are stacked sequentially according to the number of filters specified by a tunable filter parameter. This modular structure enables the network to progressively extract hierarchical features. After the convolutional blocks, the output is passed through a flattening layer and subsequently fed into a dense layer with 10 neurons and ReLU activation, followed by a softmax layer for multiclass classification. The softmax output layer assigns probabilities across the eight α-amylase concentration classes, allowing the model to select the most likely category.

### 2.6. Object Detection

To automatically localize regions of interest within the acquired images, we employed the YOLOv4-tiny object detection model, a compact and efficient variant of the YOLOv (You Only Look Once) family. YOLOv4-tiny is designed for real-time detection tasks where computational efficiency is crucial, making it suitable for deployment in portable diagnostic systems. In contrast to conventional methods that rely on fixed cropping or manual ROI selection, YOLOv4-tiny processes the entire image in a single pass, enabling the model to detect relevant objects—such as the reaction solution within a vial—directly and dynamically. This eliminates the need for pre-defined bounding boxes and allows the system to adaptively focus on the informative area of each sample. In our application, the model was trained to identify whether a saliva-containing vial is present and to precisely locate the colorimetric reaction region. This step is essential for downstream classification, as it ensures that the input to subsequent models corresponds only to the reaction area, minimizing noise from the background. The decision to use YOLOv4-tiny was motivated by its balance of detection accuracy and lightweight architecture, making it an ideal choice for integration into low-power edge devices. Furthermore, this model had already been successfully applied in a previous study to detect the reaction solution using images captured from a high-resolution smartphone camera [33]. However, in this work, we aim to evaluate its effectiveness under more constrained imaging conditions, using a low-cost embedded camera module integrated into our custom-built SCHEDA device.

### 2.7. Image Segmentation

Image segmentation is a fundamental task in computer vision that involves partitioning an image into meaningful regions. Unlike object detection, which identifies objects using bounding boxes, segmentation provides pixel-level accuracy, allowing for precise localization of the object of interest. In this study, accurate segmentation is particularly important, as the classification of color intensity must be performed exclusively on the reaction solution within the vial, ensuring that external background pixels do not introduce noise into the estimation of the α-amylase concentration. To achieve this, we employ YOLOv8, an advanced deep learning model that integrates both object detection and instance segmentation within a single architecture. Unlike traditional segmentation models that leverage separate object detection and segmentation networks, YOLOv8 streamlines the process by simultaneously identifying and segmenting objects with high precision, ensuring that classification is based only on the relevant pixels. It builds on the success of previous YOLO architectures by incorporating improved anchor-free detection, adaptive spatial feature fusion, and efficient network design, resulting in fast inference and high segmentation accuracy. These enhancements should make YOLOv8 well-suited for real-time biomedical applications, where efficiency and precision are equally critical. By utilizing YOLOv8’s segmentation capability, we ensure that the model learns to distinguish the reaction solution without requiring additional post-processing steps, thus simplifying the overall pipeline. By using this model, we aimed to evaluate whether image segmentation-based localization could enhance the overall classification accuracy without adding significant computational overhead.

## 3. Experimental Setups

### 3.1. SCHEDA: A Custom Imaging Device for Saliva Analysis

To ensure standardized and reproducible image acquisition, we developed SCHEDA (Smart Colorimetric Hardware for Enhanced Diagnostic Application), a portable, self-contained imaging device designed for point-of-care applications. The device integrates a high-efficiency microcontroller (i.e., ESP32-S3-WROOM), an OV2640 camera module (OmniVision Technologies, Santa Clara, CA, USA), an LED-based illumination system, and a dedicated sample positioning mechanism, all housed within a light-isolated 3D-printed black enclosure to eliminate external light interference, reduce reflections, and enhance imaging consistency. The ESP32-S3-WROOM serves as the processing unit, handling image capture, LED illumination control, Bluetooth communication, and overall device management. Firmware was developed in C++ using the ESP-IDF framework (version 5.1.2), allowing for efficient control and wireless communication with a companion smartphone application. The device communicates with a smartphone application via Bluetooth Low Energy (BLE) 5.0, supporting a maximum transmission rate of 2 Mbps. The estimated transfer time for an image is approximately 2 s due to BLE protocol overhead, processing constraints, and depending on connection quality.

The imaging system consists of an OV2640 camera module with a fixed-focus lens, supporting a maximum resolution of 1600 × 1200 pixels (UXGA) and multiple image formats, including JPEG, RGB565, and YUV422. To ensure consistent illumination, eight white LEDs (4500K) are positioned around the camera, eliminating unwanted shadows and reflections. A drawer-based sample holder maintains a fixed camera-to-sample distance, preventing focus variability and ensuring uniform image capture. For portability, SCHEDA operates on a 2600 mAh Li-ion battery, enabling standalone operation. Power management is handled via an integrated charging circuit with USB-C input, allowing for seamless recharging, and a hardware power switch enables safe toggling between OFF and ON states. Physically, the device measures 94 mm × 94 mm × 89 mm and weighs approximately 240 g, making it compact and lightweight for point-of-care use.

**Figure 3:**
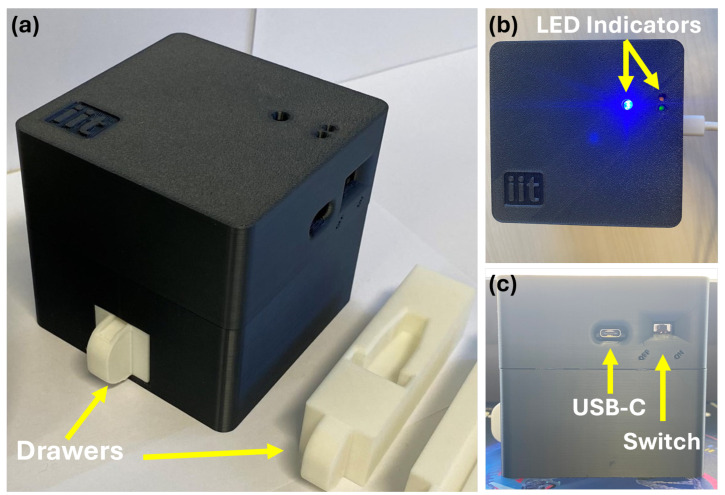
Images of the SCHEDA device. (**a**) Perspective view showing the main enclosure and removable drawers designed to hold reaction vials. (**b**) Top view with LED indicators. (**c**) Side view showing the USB-C charging port and power switch.

To support user interaction, the top panel includes three LED indicators: one for Bluetooth activity and transmission, and two additional LEDs that display battery and charging status. The overall device enclosure, shown in Figure 3, was designed to balance functionality, portability, and ease of fabrication for future integration into diagnostic workflows.

### 3.2. Dataset Preparation and Annotation

**Figure 4:**
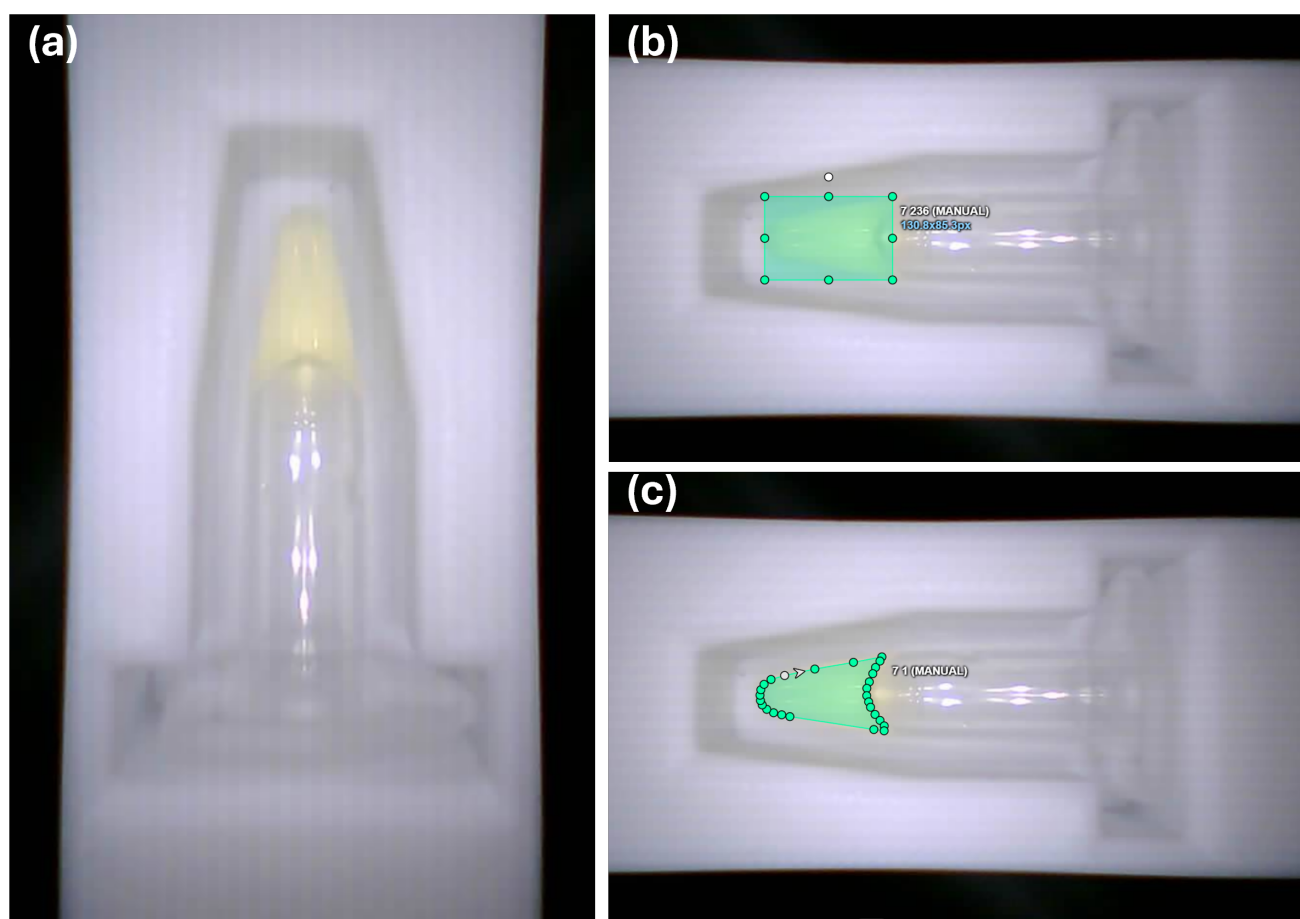
Example of dataset annotations. (**a**) Original image acquired by the device camera showing a vial positioned in the drawer, along with the image resolution. (**b**) Bounding box annotation used for YOLOv4-CNN training. (**c**) Pixel-wise segmentation mask used for YOLOv8 training.

To train the models using the two approaches (the YOLOv4-CNN and YOLOv8), we utilized the augmented dataset of 1024 images, consisting of vials with varying color intensities corresponding to different α-amylase concentration levels. An example of an original image acquired by the device camera, illustrating a vial positioned inside the drawer, is shown in Figure 4a. Each image was assigned one of eight classes, representing distinct sAA concentration levels of the reaction solution.

For the YOLOv4-CNN baseline, the dataset was annotated using bounding boxes, marking the saliva vial in each image to ensure accurate localization of the region of interest (ROI) before classification. Annotation was performed using CVAT (Computer Vision Annotation Tool) [57], which allowed for precise manual bounding box placement to define the detected object’s spatial boundaries. An example of a bounding box annotation is shown in Figure 4b. The YOLO-format annotation files exported from CVAT were directly used for training the YOLOv4-CNN model without requiring any additional preprocessing or conversion steps.

Additionally, for the proposed YOLOv8 segmentation approach, annotations were also performed using CVAT. But, in this case, pixel-wise segmentation masks were generated for each saliva sample. This method provided a more detailed and precise representation of the sample’s shape and boundaries, allowing YOLOv8 to directly classify the sample region without requiring a separate detection step (see Figure 4c).

Since the primary objective of the models is to detect and classify the reaction solution, no separate class was introduced for an empty drawer. Instead, if no reaction solution is detected within the drawer, the system assumes that no vial is present. This approach ensures that both YOLOv4 and YOLOv8 implicitly determine vial presence based on the absence of the segmented solution, avoiding the need for an additional “No vial” class.

To convert the segmentation masks into YOLOv8-compatible labels, we used a custom Python script that extracted the contours of each binary mask and converted them into polygon annotations. These polygons were normalized and saved in the YOLO format, where each object was assigned the appropriate class number. This preprocessing step ensured that the pixel-wise annotations generated using CVAT could be seamlessly integrated into the YOLOv8 training pipeline. By comparing these two approaches, we aim to demonstrate that YOLOv8’s direct segmentation-classification model outperforms the YOLOv4-CNN two-block pipeline both in terms of classification accuracy and overall computational efficiency.

### 3.3. Algorithms Hyperparameters

The training procedure included model selection through a grid search strategy, where multiple combinations of architecture-level hyperparameters were systematically evaluated. For each candidate configuration, validation accuracy was monitored throughout the training phase, and the best-performing model was retained for final testing.

As mentioned, a one-dimensional Convolutional Neural Network (1D-CNN) was employed, and its architecture was optimized using the following hyperparameters:

- Number of convolutional layers: 2 to 4 (filter configurations included (5,5), (8,8), (12,12), (12,24), (5,8,16), (5,5,5), and (8,8,8));
- Kernel sizes: Ks={6,8,12,16};
- Dropout rate: 20%.

In this context, each filter configuration defines the number of 1D convolutional kernels applied at each layer. For instance, setting f=(5,8,16) implies three sequential convolutional blocks, with 5, 8, and 16 filters, respectively. Each block includes a 1D convolutional layer followed by ReLU activation, dropout, and average pooling. The filter count was either fixed or increased progressively to allow for deeper feature abstraction. This structure enables the model to learn hierarchical patterns while maintaining reduced computational complexity compared to 2D CNNs. All candidate configurations were trained and evaluated on the validation set, and the model that achieved the highest validation accuracy was selected for downstream testing.

The YOLOv4-tiny model was configured using the same hyperparameter settings adopted in our previous study [33], as they were found to be effective for reaction solution detection tasks. Specifically, the training parameters were as follows:

- Batch size and subdivisions: Set to 64 and 16, respectively, to balance training efficiency and GPU memory usage.
- Input resolution: 416 × 416 pixels, chosen to balance detection accuracy with computational cost.
- Learning rate: Initialized at 0.001 with a burn-in of 1000 iterations to allow for stable weight updates.
- Maximum iterations: Set to 6000 to ensure sufficient training convergence.

For the proposed YOLOv8-based segmentation-classification model, the hyperparameters were optimized using a grid search approach to determine the best-performing configuration. The following hyperparameters were systematically explored:

- Initial learning rate ($lr_{0}$): 0.001,0.005,0.01.
- Batch size: 8,16, adjusted based on GPU memory availability.
- Momentum: 0.9,0.937.
- Optimizer: Stochastic Gradient Descent (SGD) and AdamW.

A grid search was performed, iterating over all possible combinations of the above hyperparameters. The dataset configuration was defined in conFigureyaml, specifying eight classes corresponding to α-amylase concentration levels (labeled from 0 to 7).

The model’s performance was evaluated on a validation set, selecting the configuration that minimized validation loss. The best model was then tested on a separate test set, ensuring robust generalization.

### 3.4. Training Strategy

#### 3.4.1. YOLOv4-CNN Approach

The training of the YOLOv4-tiny model was carried out using the annotated dataset described in Section 3.2, where bounding boxes were used to label the regions of interest containing the reaction solution. The model was trained on Google Colab using the Darknet framework with GPU acceleration. Throughout the training process, model performance was evaluated using the mean Average Precision (mAP) metric to monitor convergence and detection accuracy.

After completing the training, a 16-bit quantization step was applied to reduce the model’s size and optimize it for deployment on resource-constrained platforms. The quantized YOLOv4-tiny model was then used to process the original image dataset, extracting the detected regions of interest (ROIs). Each detected ROI, corresponding to the reaction solution, was cropped and resized to a standardized resolution of 100 × 70 pixels, creating a new preprocessed dataset referred to as *yolov4_data*.

To prepare the data for classification, only the red channel was retained from each cropped image, based on prior analysis showing that red intensity carries the most discriminative information for colorimetric analysis (see Section 2.2). As part of our evaluation, we initially trained a 2D CNN on the full RGB images following the same approach as in our previous study [33]. For both approaches, whether using RGB or red-channel-only input, the images were normalized using a MinMaxScaler to scale pixel values between 0 and 1, ensuring uniform feature distribution and enhancing training stability.

A CNN classifier was subsequently trained using the *yolov4_data* dataset to solve the 8-class color intensity classification problem. The dataset was randomly divided into three subsets: approximately 56% for training (576 images), 24% for validation (248 images), and 20% for testing (200 images). This stratified split ensured class balance and allowed for a fair evaluation of the classifier’s generalization performance. The overall pipeline was designed to assess whether the YOLOv4-CNN approach could obtain a good classification accuracy of α-amylase concentration levels and to compare the effectiveness of using the full RGB images versus red-channel-only input for feature extraction.

#### 3.4.2. YOLOv8 Approach

For the YOLOv8-based segmentation-classification pipeline, we employed the YOLOv8m-seg model, an instance segmentation network pre-trained on the COCO dataset. The model was fine-tuned on our annotated dataset (Section 3.2), where each image was labeled with a pixel-wise segmentation mask and an associated α-amylase concentration class ranging from 0 to 7. To ensure a fair comparison with the YOLOv4-CNN approach, the same dataset partitioning was used: 56% for training, 24% for validation, and 20% for testing, maintaining class balance across all subsets.

Training was conducted on Google Colab with GPU acceleration using the Ultralytics YOLOv8 framework, utilizing YOLOv8m-seg, a medium-sized segmentation model pre-trained on COCO. Prior to training, a grid search was conducted over multiple combinations of learning rate, batch size, momentum, and optimizer type to identify the best-performing configuration (see Section 3.3).

The input images were resized to 1024 × 1024 pixels to ensure high segmentation precision and classification accuracy. Each model configuration was trained for 15 epochs, a range found to be sufficient based on preliminary experiments to avoid overfitting while ensuring convergence. The model achieving the lowest validation loss was selected for final evaluation on the test set.

## 4. Results and Discussion

This section presents the performance evaluation of the proposed approaches for classifying salivary α-amylase concentration levels from colorimetric images. Three distinct models were assessed: (1) a baseline YOLOv4-CNN pipeline trained on RGB images. (2) YOLOv4-CNN with a 1D CNN architecture trained using only the red channel. (3) a proposed YOLOv8-based segmentation-classification model that performs pixel-level segmentation and classification in a single step. The performance of each model was quantified using accuracy scores and visualized through confusion matrices, displaying both absolute predictions and per-class percentages. All models were evaluated on the same test set comprising 25 images per class (200 total), ensuring a consistent and fair comparison.

**Figure 5:**
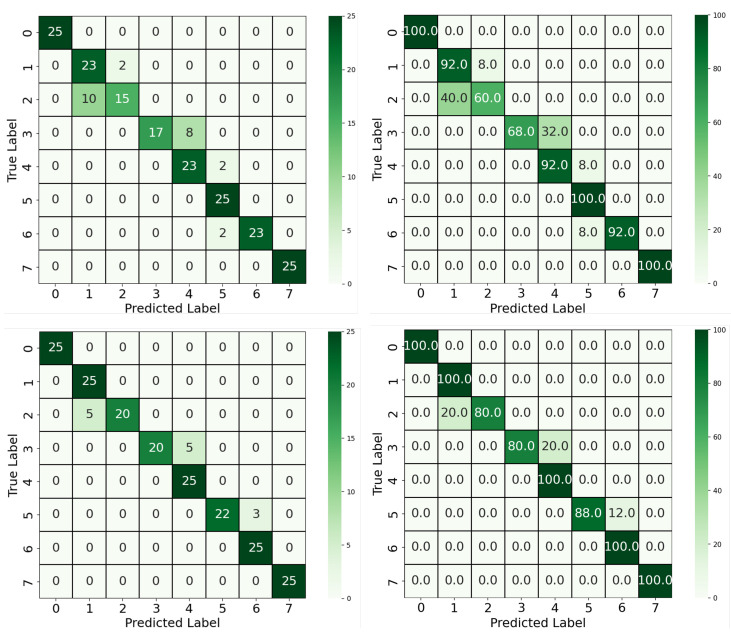
Confusion matrices for the YOLOv4-CNN approach. Top: RGB input; Bottom: Red-channel-only input. Left panels show sample counts; right panels show percentage accuracy per class.

We first compared the two-stage YOLOv4-CNN pipeline using two input configurations: full RGB images and red-channel-only images. As shown in Figure 5, the RGB-based approach achieved an accuracy of 88%, with some confusion observed between neighboring classes, particularly between Classes 1 and 2 and Classes 3 and 4. These confusions can be attributed to the visual similarity in overall color tones when using all three channels, which may dilute the discriminative signal. In contrast, the red-channel-only approach yielded a notable improvement, achieving 93.5% accuracy. As supported by the color analysis in Section 2.2, the red channel offers the most separable signal across concentration levels. This improvement confirms that focusing the classifier on the most relevant color channel can enhance performance while also reducing model complexity. Moreover, five out of eight classes achieved 100% classification accuracy using the red-channel input, compared to only three classes in the RGB-based model, further highlighting the discriminative power of the red spectrum in this colorimetric classification. Beyond classification accuracy, this approach offered architectural and computational advantages. By reducing the dimensionality of the input to a single color channel and adopting a 1D convolutional architecture, we achieved a leaner model that is both more computationally efficient and easier to interpret.

**Figure 6:**
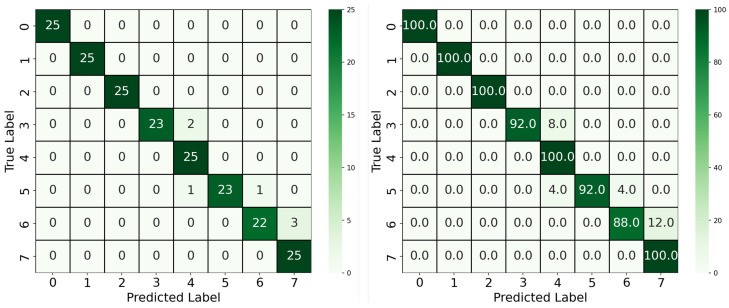
Confusion matrix for the YOLOv8 segmentation-classification model. Left: Absolute counts; Right: Per-class percentage accuracy.

Furthermore, building upon the YOLOv4-CNN framework, the YOLOv8 model was introduced to streamline the pipeline by combining segmentation and classification into a single unified architecture. The model was trained using pixel-wise segmentation masks and concentration-level class labels, as described in Section 3.2. The segmentation allowed the model to focus exclusively on the reaction region, discarding irrelevant background pixels. As illustrated in Figure 6, this approach achieved the highest overall performance, with a classification accuracy of 96.5%. Only minor misclassifications occurred between adjacent classes, such as Classes 6 and 7, which likely share subtle color differences. The significant improvement over the two-block pipeline demonstrates the benefit of precise ROI extraction via segmentation, as well as the advantages of joint learning of localization and classification.

Beyond accuracy, we evaluated all models using macro-averaged precision, recall, and F1-score to account for class-wise performance in this multiclass classification task. These metrics are particularly appropriate given the balanced test set, as they assign equal weight to each class regardless of frequency. As summarized in Table 2, the YOLOv4-CNN model trained on RGB images achieved a precision of 0.8979, recall of 0.8800, and F1 score of 0.8778. Moreover, using the red-channel-only variant improved performance across all three metrics, reaching a precision of 0.9449, recall of 0.9350, and F1 score of 0.9344. On the other hand, the proposed YOLOv8-based segmentation-classification model achieved the highest overall performance with precision, recall, and F1 score of 0.9678, 0.9650, and 0.9650, respectively. These results confirm that the YOLOv8 architecture, which integrates segmentation and classification, not only improves accuracy but also provides superior class-level consistency and robustness.

Additionally, we also compared the memory footprint and operational complexity of the two pipelines. As summarized in Table 3, the total model size of the YOLOv4-CNN approach was around 11.78 MB when both YOLOv4-tiny and the CNN classifier were quantized to float16 precision. In contrast, the unified YOLOv8m-seg model used in the proposed pipeline occupied 52.2 MB in float16 format, i.e., approximately four times larger. Despite its smaller size, the YOLOv4-CNN pipeline introduces additional architectural and computational complexity. After YOLOv4 detection, preprocessing is required to extract the ROI, followed by red-channel extraction and normalization before being fed to the CNN classifier. This two-stage workflow involves inter-model dependencies and intermediate data transformations, which may increase latency and make on-device deployment more challenging, especially on resource-constrained platforms. On the other hand, the YOLOv8-based approach streamlines the process by combining instance segmentation and classification into a single model. Original input images are directly processed without the need for intermediate ROI extraction or channel manipulation. This not only simplifies the overall pipeline but also enables faster inference, easier integration into mobile applications, and improved maintainability.

These considerations suggest that although YOLOv8m-seg has a larger memory footprint, its architectural simplicity and operational efficiency make it a more scalable and deployable solution for smart point-of-care diagnostic systems.

In addition to software performance, this study validates the hardware capabilities of the SCHEDA imaging system. Combined with the mobile application, SCHEDA enabled reliable, high-quality image acquisition under consistent lighting conditions across all samples. The Bluetooth-enabled interface allowed any smartphone to act as the processing unit, decoupling model inference from variations in phone camera optics or lighting.

To ensure imaging consistency and mitigate potential artifacts, the SCHEDA device was designed with a fixed internal configuration, including a mounted camera, uniform LED illumination, and a stable sample holder within a light-isolated chamber. This mechanical design inherently ensures consistent optical geometry and eliminates external lighting interference. During initial development, blank vials and color reference cards were imaged to validate illumination uniformity and verify the absence of visible gradients or reflections. Moreover, multiple SCHEDA units were independently fabricated and tested under identical conditions to assess reproducibility. All units produced consistent imaging results, confirming the robustness and generalizability of the hardware design.

Together, the integration of SCHEDA with the proposed YOLOv8 model enabled a fast, accurate, and fully mobile-ready solution for classifying α-amylase concentration levels. The trained model was successfully exploited within a custom mobile application and tested online on a Xiaomi 11 Lite smartphone. Real-time inference yielded classification results consistent with offline evaluation, confirming the model’s robustness and its suitability for real-time, point-of-care diagnostic applications.

## 5. Conclusions

In this study, we presented a robust, mobile-ready diagnostic framework for classifying salivary α-amylase concentration levels using a deep learning-based analysis of colorimetric reactions. The proposed system integrates a custom-designed imaging platform (SCHEDA) with advanced AI pipelines to address the limitations of conventional laboratory assays and colorimetric tests, particularly in terms of portability, environmental sensitivity, and user subjectivity.

We compared two AI-based classification strategies for solving an 8-class classification problem, corresponding to distinct levels of α-amylase concentration. The first strategy employed a sequential YOLOv4-CNN pipeline, while the second utilized a unified YOLOv8 segmentation-classification approach. Our results demonstrated that extracting the red channel from the reaction solution images significantly improved classification accuracy over the full RGB input. Specifically, using red-channel-only images in the YOLOv4-CNN pipeline, we achieved an accuracy of 93.5%, surpassing the 88% obtained with RGB images. The performance further improved with the YOLOv8 model, which reached 96.5% accuracy by leveraging pixel-wise segmentation to isolate the reaction region before classification.

While the YOLOv4-CNN pipeline featured a smaller model footprint (11.78 MB) compared to YOLOv8 (52.2 MB), it required multiple intermediate steps, including object detection, ROI cropping, red-channel extraction, and normalization. In contrast, the YOLOv8 model offered a streamlined end-to-end solution with reduced pipeline complexity and easier deployment on mobile platforms. Additionally, we validated the practicality of the SCHEDA device, which ensures consistent image acquisition under controlled lighting and integrates seamlessly with a smartphone via Bluetooth. The full pipeline was successfully deployed in a mobile application and tested on a Xiaomi 11 Lite smartphone, where real-time inference yielded results in line with offline evaluations. This confirms the suitability of the system for field-deployable, point-of-care applications.

Overall, this work demonstrates the potential of combining low-cost, standardized hardware with efficient AI models for non-invasive biomarker quantification. The proposed solution supports scalable and accurate classification of salivary α-amylase levels, offering a promising tool for stress monitoring and broader diagnostic applications in mobile healthcare.

## Figures and Tables

**Table 1:** Dataset representation in terms of α-amylase concentration and number of samples per class before and after data augmentation.

	α-Amylase Concentration (U/mL)	Nb. of Samples Before Aug.	Nb. of Samples After Aug.
**Class 0**	0–15	(57)	(128)
**Class 1**	15–30	(60)	(128)
**Class 2**	30–45	(64)	(128)
**Class 3**	45–60	(61)	(128)
**Class 4**	60–80	(58)	(128)
**Class 5**	80–100	(60)	(128)
**Class 6**	100–120	(62)	(128)
**Class 7**	>120	(58)	(128)

**Table 2:** Macro-averaged classification metrics for each model on the test set.

Model	Accuracy	Macro Precision	Macro Recall	Macro F1
YOLOv4-CNN (RGB)	88.00%	0.8979	0.8800	0.8778
YOLOv4-CNN (Red only)	93.50%	0.9449	0.9350	0.9344
YOLOv8 + CNN	96.50%	0.9678	0.9650	0.9650

**Table 3:** Comparison of model sizes (float16 precision) between YOLOv4-CNN and YOLOv8 pipelines.

	YOLOv4 -Tiny	CNN	YOLOv8m -Seg	Total Model Footprint
**YOLOv4-CNN**	11.5 MB	0.28 MB	-	11.78 MB
**YOLOv8**	-	-	52.2 MB	52.2 MB

## Data Availability

The original data presented in this study are openly available in the GitHub repository at https://github.com/YoussifAmin/Alpha_Amylase (accessed on 29 May 2025).
